# Comparison of the effectiveness and outcome of microendoscopic and open discectomy in patients suffering from lumbar disc herniation

**DOI:** 10.1097/MD.0000000000016627

**Published:** 2019-12-16

**Authors:** Ram Ishwar Yadav, Ling Long, Cao Yanming

**Affiliations:** The Second Affiliated Hospital of Guangzhou Medical University, Department of Orthopaedics, Guangzhou, China.

**Keywords:** lumbar disc herniation, micro-endoscopy, open discectomy

## Abstract

**Background:**

The purpose of our study is to compare the outcomes and effectiveness of MED vs OLD for lumbar disc herniation.

**Objectives:**

To identify the functional outcomes in terms of ODI score, VAS score complications in terms of intraoperative blood loss, use of general anesthesia, and morbidity in terms of total hospital stay between MED and OLD.

**Methods:**

In our randomized prospective study we analyzed 60 patients with clinical signs and symptoms with 2 weeks of failed conservative treatment plus MRI or CT scan findings of lumbar disc herniation who underwent MED and OLD. The study was undertaken from November 2017 to January 2019 at Guangzhou Medical University of Second Affiliated Hospital, department of orthopedic surgery in spinal Unit, Guangzhou, China. Patients were divided into 2 groups i.e. who underwent MED group and the OLD group then we compared the preoperative and postoperative ODI and VAS score, duration of total hospital stay, intraoperative blood loss, and operation time.

**Results:**

We evaluated 60 patients. Among them, 30 underwent MED (15 female and 15 male) and 30 underwent OLD 14 male 16 female. Surgical and anesthesia time was significantly shorter, blood loss and hospital stay were significantly reduced in patients having MED than OLD (<0.005). The improvement in the ODI in both groups was clinically significant and statistically (*P* < .005) at postoperative 1st day (with greater improvement in the MED group), at 6 weeks (*P* > .005), month 6 (>0.005) statistically no significant. The clinical improvement was similar in both groups. VAS and ODI scores improved significantly postoperatively in both groups. However, the MED group was superior to the OLD group with less time in bed, shorter operation time, less blood loss which is clinically and statistically significant (*P* < .05).

**Conclusions:**

The standard surgical treatment of lumbar disc herniation has been open discectomy but there has been a trend towards minimally invasive procedures. MED for lumbar spine disc herniation is a well-known but developing field, which is increasingly spreading in the last few years. The success rate of MED is about approximately 90%. Both methods are equally effective in relieving radicular pain. MED was superior in terms of total hospital stay, morbidity, and earlier return to work and anesthetic exposure, blood loss, intra-op time comparing to OLD. MED is a safe and effective alternative to conventional OLD for patients with lumbar disc herniation.

## 1 Introduction

Low back pain (LBP) has become one of the most serious public health problems, with a lifetime prevalence as high as 84% and the prevalence of chronic low back pain is about 23%, with 11% to 12% of the population being disabled by low back pain.^[1]^ In China, the prevalence of lumbar disc herniation is high in civil servants (44.8%).^[2]^ To date, the factors that eventually cause pathological progression have not been determined. However, along with recent economic development, living, environmental, and working conditions have substantially changed in China.^[16]^ Lumbar disc herniation is one of the most common spinal degenerative disorders leading to LBP associated with radiculopathy.^[3–6]^ On the other hand, some studies found that disc herniation was actually common in asymptomatic people as well.^[5,7,8]^ Inflammatory response has been acknowledged to be important in the process of disc degeneration^[9–11]^ and may play an important role in pain generation.^[12,15]^

**Figure 1:**
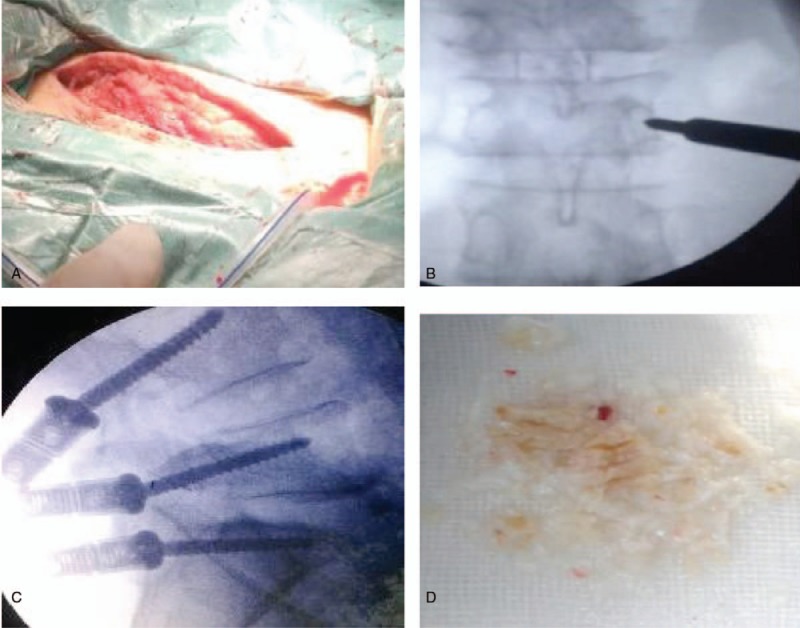
Sagittal T1-weighted MR images (left) and sagittal STIR MR images (right). (a and b) - Low signal intensity (SI) on T1-weighted images and high SI on STIR images of the endplates at L5-S1 related to a type I endplate change, (c and d) – High signal intensity on T1-weighted images and low signal intensity on STIR images of the endplates at L4-L5 related to type II endplate change.

**Figure 2:**
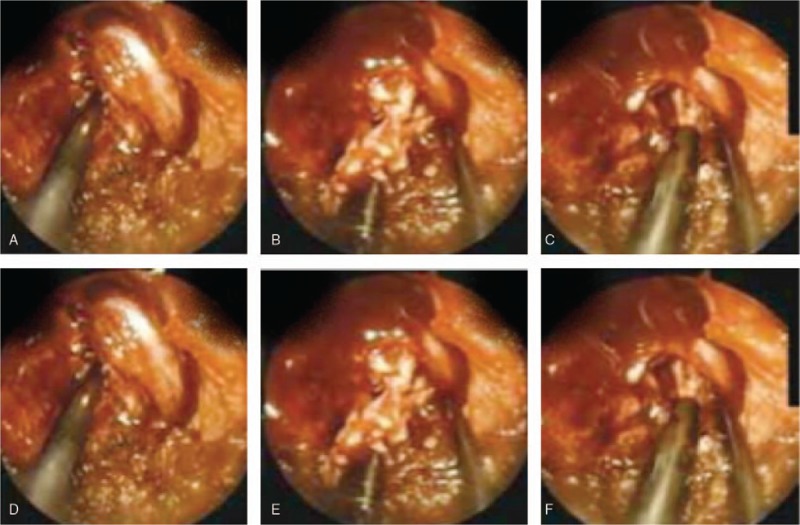
MR images showing the compression at the level of the foramen or extra-foraminal. At each level, specific pathology can be seen, but there is a lot of overlap. For instance, a disc can herniate and cause nerve compression at the level of the disc, but can also migrate to a lower level and compress the nerve in the lateral recess or move upward and cause compression at the level of the foramen or extra-foraminal. In patients with facet arthrosis, the bony spurs can move medially and narrow the lateral recess or move upward and narrow the foramen. When there is extreme facet arthrosis bilaterally, it can cause stenosis of the spinal canal and compress all the nerve roots at that level.

**Figure 3:**
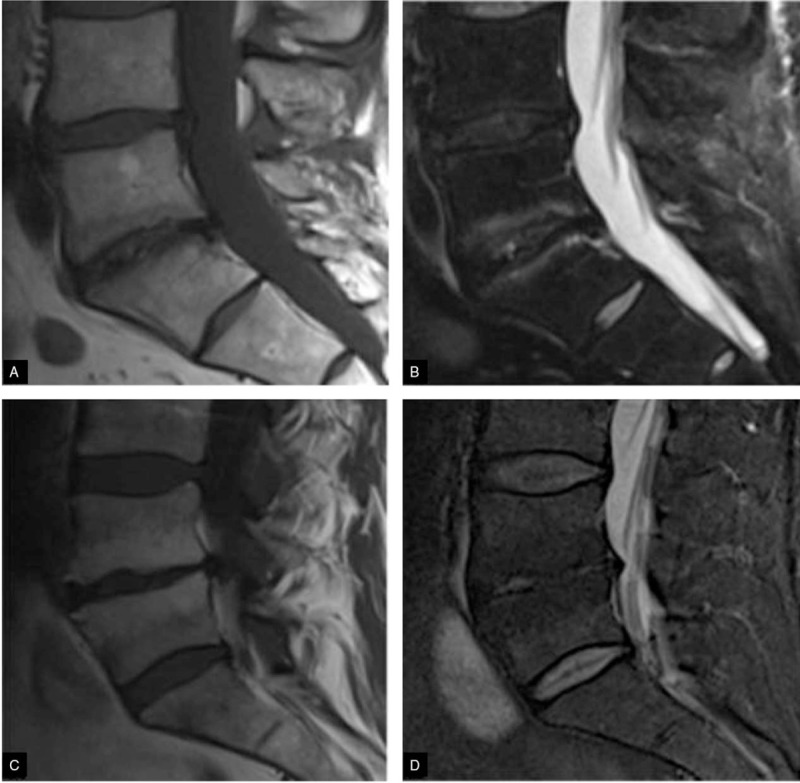
MR images of lumbar disc at the different levels from 1 to 4. The findings at the different levels 1 to 4 are: A At the level of the disc, there is minimal spinal canal narrowing by bulging of the disc and facet arthrosis. (B) At this level, there is severe spinal canal stenosis due to bulging disc and facet arthrosis. There is also an annular tear (high signal) through which the disc herniates (yellow arrow) (C) At the level of the lateral recess, there is a focal herniation of disc material compressing the L5 nerve (yellow arrow). This is called an extrusion because the distance between the edges of the disc material is greater than the distance at the base. “And (D) Compressed” L5 nerve (blue arrow) within the lateral recess. The herniated disc has migrated caudally and is seen as an oval structure anterior to the nerve. The herniated disc is uncontained, i.e. not covered by fibers of the annulus.

Intervertebral disc Annulus Fibrous (AF) tear is another important factor related to disc degeneration and pain generation.^[13,14,18–21]^ Previous studies demonstrated that AF tear detected by histology and MRI in the patient with LBP could be considered a reliable marker for a painful disc.^[13,14,19,20]^ However, controversial results were also reported^[22,23]^ indicating that AF tear alone may not be sufficient to cause LBP arising from a degenerative disc. One comprehensively accepted an explanation of AF tears causing LBP is that it could enhance the transportation of macromolecules from the NP to AF and in growth of nerve fibers into internal AF or NP.^[13,14,24,25]^ Thus, we hypothesize that under the circumstances of disc degeneration, overexpressed inflammatory mediators acting as pain stimuli transport from the NP to external AF or even the periphery of AF, and interact with the nociceptive receptors that are usually located around the periphery of AF and have grown into the AF even NP through tears consequently causing LBP. Morphological characteristics, namely the arrangement of the annular fiber bundles, seem to contribute to the propensity for disc herniation on the posterior aspect of the disc. A disc bulge is a symmetrical extension of the disc beyond the endplates (Fig. 1), whereas a protrusion is a focal area of extension still attached to the disc. An extruded fragment is 1 that is no longer connected to the disc (Fig. 3) and a sequestred fragment is contained within the PLL. At each level specific pathology can be seen, but there is a lot of overlap. For instance a disc can herniate and cause nerve compression at the level of the disc, but can also migrate to a lower level and compress the nerve in the lateral recess or move upward and cause compression at the level of the foramen or extra-foraminal. In patients with facet arthrosis the bony spurs can move medially and narrow the lateral recess or move upward and narrow the foramen. When there is extreme facet arthrosis bilaterally, it can cause stenosis of the spinal canal and compress all the nerve roots at that level (Fig. 2 and).

## 2 Aims and objectives

### 2.1 “General objectives”

- The aim of the study is comparative effectiveness of microendoscopic vs open lumbar discectomy for lumbar disc herniation is to compare the effectiveness of micro endoscopic discectomy vs open discectomy in terms of its outcome.
- And to compare hospital management of patients with Lumbar disc herniation in the above 2 groups.

### 2.2 Specific objectives

- To compare the most effective methods of surgery among OLD and MED in patients with lumbar disc herniation in terms of total blood loss, the number of hospital stays required, operation time required, VAS and ODI scores.

## 3 Materials and methods

### 3.1 Study design and methods

We divided the total of 60 patients into 2 groups ie. MED and OLD group, 30 in each group who underwent MED and OLD for lumbar disc herniation and did a randomized prospective study. The study was conducted in Spinal unit at Guangzhou Medical University of Second affiliated Hospital, Guangzhou, Guangdong province, China from November 2017 to January 2019.

We compared the preoperative VAS and ODI with postoperative VAS and ODI score at 1st day, 6weeks, and 6 months. We also compared the intra-operative blood loss, the total duration of hospital stay and operation time.

### 3.2 Ethics and consent

This study is part of a quality management program on anonymized patients; therefore, no institutional review board approval is required.

### 3.3 Inclusion and exclusion criteria

Inclusion criteria:

- Age 20 to 90 years.
- Persistent radicular pain lasting for more than 6 to 8 weeks.
- Disc herniation confirmed by MRI Single level herniation.
- Adjacent bi-segmental herniation.
- Desiccated disc with body root.
- Entrapment/lateral canal stenosis.
- Unilateral herniation was larger than 1/3 of the spinal canal diameter with concomitant lateral recess stenosis or “equestration.”

Exclusion criteria:

- Less than 2 level disc herniation.
- Cauda equina syndrome.
- Spondylolytic or degenerative spondylolisthesis.
- Spinal canal stenosis.
- Pregnancy.
- Severe somatic or psychiatric illness.

## 4 Diagnosis

### 4.1 Radiological findings

XXX

### 4.2 Operative procedure

LDH is a common disease and lumbar discectomy is the most common surgical procedure carried out for patients with low back pain and leg symptoms. Although most researchers are focusing on surgical techniques during operation.^[17]^ The success rate of lumbar discectomy is about 70% to 90%.^[26,27]^

#### 4.2.1 Open lumbar discectomy

**Figure 4:**
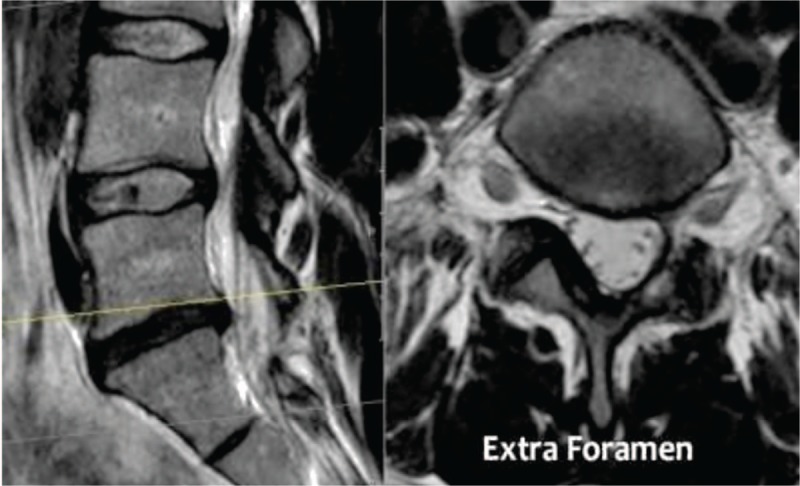
Open lumbar discectomy with pedicle screw fixation. (A) Mid line incision. (B) Image intensifier radiographs of pedicle screw insertion point anterior-posterior view. (C) Intraoperative pedicle screw fixation. (D) Removal of disc materials.

It is a posterior instrumentation procedure, in which we make a longitudinal midline skin incision usually between 3.5 and 5 cm. We also estimate the required screw length from the preoperative magnetic resonance images. We make a deep wound through the muscle fascia (Fig. 4 A). The gantry is adjusted to parallel the trajectory of fluoroscopy and upper-end plate of the vertebra of interest and is rotated to position, the spinous process midway between the pedicles of the vertebra of interest. We use a single shot method, we introduce our designed instrument into the wound until the tip made contact with the bone and confirmed that the instrument tip met the midpoint of the lateral border of the pedicle shadow on the fluoroscopy image (Fig. 4 B). That position is the entry point of our pedicle screw. Usually, it is the step that required fluoroscopy shots the most. Light hammering is used to introduce the Kirschner wire tip to break the cortical bone at the entry point. To avoid a too caudal or too cranial insertion of the screw, we make our instrument trajectory parallel to the upper-end plate of the vertebra of interest. The only decision that is to be made is the entry point of the pedicle screw and screw trajectory on the AP view of fluoroscopy. The screw trajectory on the AP view is an important indicator of whether the screw is able to penetrate the inner wall of the pedicle. During the preparation for pedicle insertion, the direction and length of the projection line (PL) of the slender portion of the awl is inspected. After determining the entry point and the instrument direction (Fig. 4C), we screw the instrument slowly into the target pedicle. The shallow screw thread at the instrument tip made the purchasing power too weak to break the cortex bone, and this keeps the instrument inside the pedicle. We suggest another fluoroscopy AP shot to confirm the trajectory that provides surgeons with information on the depth of the instrument. After the pedicle is cannulated, the remaining parts of the procedure are similar to the other pedicle screw insertion. A spinal needle is then placed into an interspace and an x-ray is taken to identify the spinal level. The fascia is incised in a slightly arcuate manner in order to preserve the interspinous ligaments. The paraspinous muscles are then detached from the spinous processes, laminae, and the medial facet. Care is taken to dissect in the subperiosteal plane to avoid bleeding and undue trauma to the muscles. This is done with a sharp periosteal elevator and monopolar electrocautery. A sponge can then be guided over the bony surfaces with the periosteal elevator to clean any residual muscle left on the lamina. Remaining muscle obscuring the ligamentum flavum is removed with a Leksell or pituitary rongeur. The inferior aspect of the superior lamina, the medial facet, and the superior aspect of the inferior lamina are all thinned using a high-speed drill and/or Leksell and Kerrison rongeurs. Ligamentum flavum is remove to gain access to the epidural space. The ligamentum flavum is detached from the undersurface of the rostral lamina and then is removed in a superior to inferior manner. Ligamentum flavum detached from the rostral end of the inferior lamina are removed in an inferior to superior manner. The fibers are cut and further separated with a small dissector such as a Penfield 4 in a longitudinal fashion and subsequently removed with Kerrison punches and in conjunction with the traditional discectomy, a laminotomy is often involved to permit access to the intervertebral disc. In this procedure, a small piece of bone (the lamina) is removed from the affected vertebra, allowing the surgeon to better see and access the area of disc herniation all the herniated disc material are removed (Fig. 4 D). Finally, we rotated the fluoroscopy gantry to obtain lateral views. With lateral views, we align the depth of each pedicle screw, insert cap of the pedicle and place the rods. Hemostasis is secured and suturing is done in layers. Dressing pad is applied.

#### 4.2.2 Micro-endoscopic lumbar discectomy

**Figure 5:**
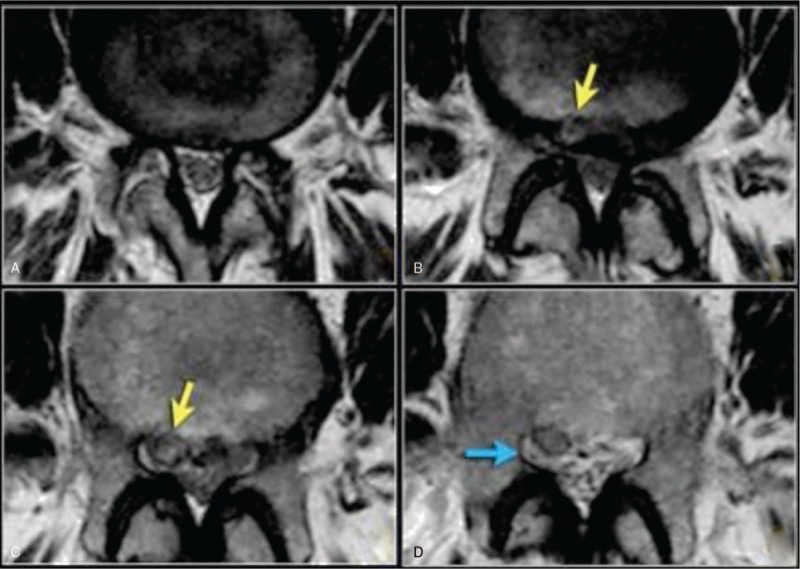
Micro-Endoscopic Lumbar Discectomy. (A) Showing position of the patient in MED. (B) T.V monitor. (C) Endoscopic views of the facet joint and ligamentum flavum, (D) the spinal cord and nerve root (E) removal of disc material, and (F) checking of movements of a nerve root and spinal cord.

EDS for lumbar spine disc herniation is a well-known but developing field, which is increasingly spreading in the last few years.^[28]^ Micro-discectomy and minimally invasive discectomy decrease surgical exposure and trauma and have success rates of approximately 90%.^[26,27]^ Spinal endoscopic techniques have evolved more slowly, because of the complex anatomy and difficult access.^[29]^ Endoscopic extraction of disc fragments become feasible, as anatomic structures can be visualized using small-calibre, high-resolution glass fiber optics. Nonetheless, the endoscopes are expensive and standardization of size is lacking.^[30]^ Minimally invasive techniques reduce postoperative morbidity and the incidence of perineural and intraneural fibrosis.^[31]^ “MED preserves” the epidural venous system^[32–36]^ and minimize the development of instability and spondyloarthropathy.^[34]^ All the procedures are done under local anesthesia. The patient is placed in the lateral position with the abdomen free and the spine flexed to open the interlaminar space (Fig. 5 A). The surgeon stood on the side of the disc prolapse, the TV monitor is at the head end facing toward the front of the surgeon (Fig. 5 B). A flexible arm assembly is attached to the operating table rail to hold the tubular retractor with an endoscope in a stable position, freeing the surgeon's hands. The surgeon uses a tubular retractor system and a microscope to guide his movements and perform the operation. At first cleaning and draping is done then a guide wire is inserted through a small incision to locate the affected disc in the spine. We use a fluoroscope which displays live X-ray images, to ensure that the surgeon lines up the route to the correct herniated disc. “The surgeon passes” a series of dilating tubes over the guide wire, pushing apart the tissue to get to vertebrae, facet joint, and ligamentum flavum (Fig. 5C). Once it has access to the herniated disc, we remove the guide wire. The tubular retractor is inserted, through which we perform the surgery, slides over the dilating tubes. Then he removes all the dilating tubes. A surgical light and small camera allow. All instruments are seen through the tube. We use special surgical instruments, passed through the tube, to clear away bone and soft tissue to gain access to affect spinal. We use a nerve retractor to gently move the nerve away from the herniated disc (Fig. 5 D). Again, then using small surgical instruments passed through the tubular retractor, we remove the herniated portion of the disc (Fig. 5 E), we clear the area enough to allow the nerve to move back into its normal position (Fig. 5 F). We withdraw the tubular retractor, which allows body tissue to close around the surgery site. Because the incision is small for an endoscopic discectomy, only a 2 suture and small bandage is applied to close the surface wound ().

**Figure 6:**
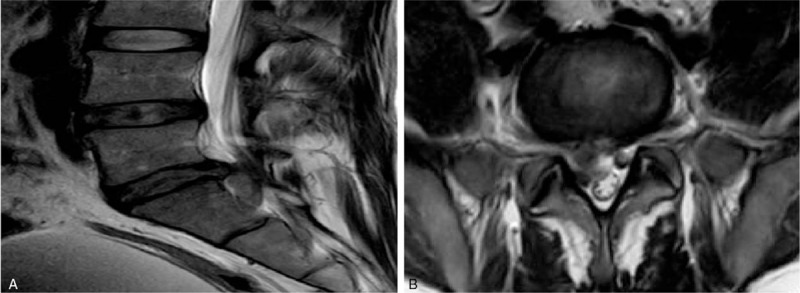
Pre-operative MRI of lumbar disc herniation. (a) Pre-operative MRI of lumbar disc herniation at L5, S1 in sagittal view. (b) Pre-operative MRI of lumbar disc herniation at L5, S1 with compression of a nerve of root left.

**Figure 7:**
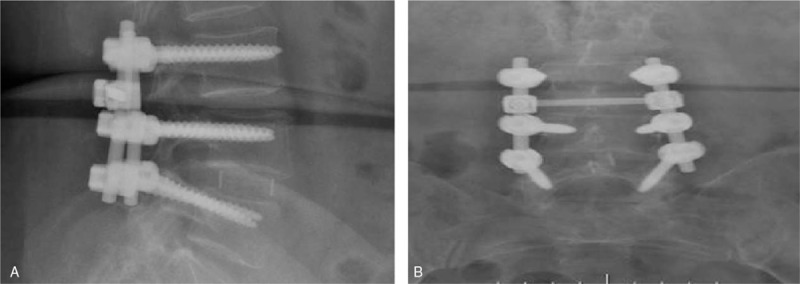
Postoperative X-ray lateral and AP view of open lumbar discectomy. (A) Postoperative X-ray lateral view of open lumbar discectomy with pedicle screw fixation of the lumbar spine at the level of L3-L5. (B) Postoperative X-ray AP view of an open lumbar discectomy with pedicle screw fixation of the lumbar spine at the level of L3-L5.

**Figure 8:**
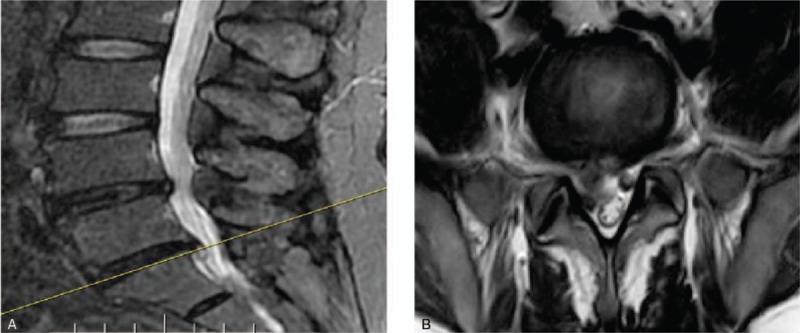
Pre-operative MRI of lumbar disc herniation at L4 L5 in sagital view and axial view. (A) Pre-operative MRI of lumbar disc herniation at L4 L5 in sagital view. (B) Pre-operative MRI of lumbar disc herniation at L4 with compression of nerve root of the left foramen (axial view).

**Figure 9:**
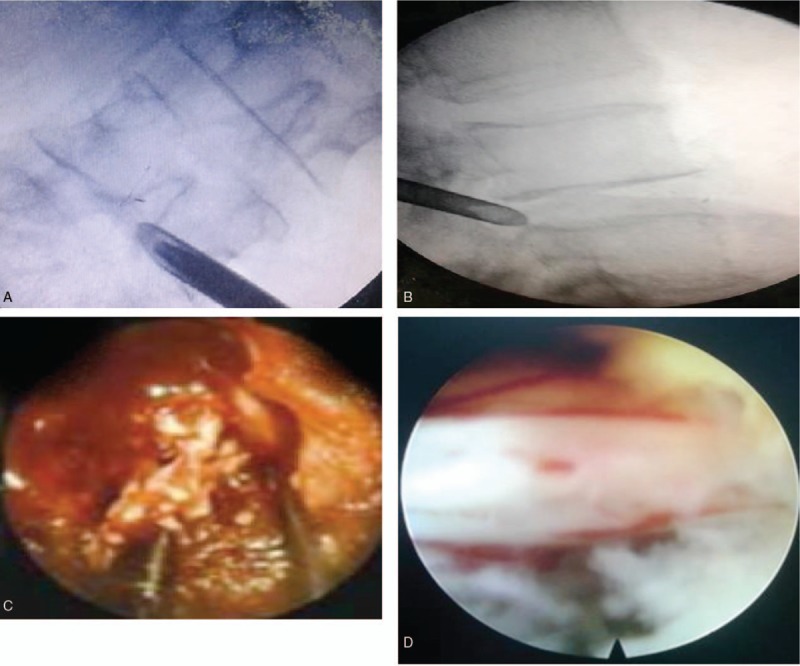
Instruments used for MED. (A) Image intensifier radiographs showing intraoperative level of disc herniation and insertion of serial dilators anterior-posterior view. (B) Radiographic image of serial dilator (lateral view). (C and D) The removal of a disc with the free release nerve root.

### 4.3 Statistical analysis

The data were analyzed by using SAS 9.21. For descriptive data frequency and the percentage was used. Mean, median, and SD were used for the parametric data while for the non-parametric bar diagram and pie chart was used. For the comparison of 2 categorical variables “Pearson” Chi-Squared test and 1 way ANOVA (analysis of variance) was used and the significance was put at *P* < .05.

## 5 Results

The demographic distribution of both groups was similar, as were the clinical and electrophysiological findings and indications for surgery. Surgical time, blood loss, and hospital stay were significantly reduced in patients having MED than open discectomy (Table 1). The improvement in the Oswestry score in both groups was significant (*P* = .005) at postoperatively day 1 but not at week 6 (*P* = .5418) and month 6 (*P* = .1498) (Table 2). The improvement in the VAS score in both groups was significant (*P* = .005) at postoperatively day 1 but not at week 6 (*P* = .083) and month 6 (*P* = .0988) (Table 3). The clinical, neurological, and electrophysiological improvement was similar in both groups. Adequate decompression was achieved, and the weight of disc material removed was similar in both groups. Both methods were equally effective in relieving radicular pain by reducing the tension on the nerve root caused by the herniated disc. The 2 groups were compared with respect to surgical time, duration of hospital stay, intraoperative blood loss, VAS score, and Oswestry score. Surgery time was longer in the OLD group than in the MED group (*P* = .006) (Table 1). The MED group was superior to the OLD group, however, with less time in bed, shorter hospital stay (*P* = .0472), less blood loss (*P* = .0001) (Table 1), lower VAS scores 1 day postoperatively (*P* = .0390) (Table 3) and lower ODI scores 1 day postoperatively (*P* = .0001). There were no significant differences in VAS or ODI scores at 6 weeks and 6 months after surgery between the 2 groups (*P* = .05) (Tables 2 and 3).

**Table 1:**
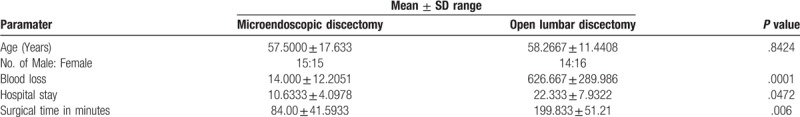
Patient demographics and outcomes.

**Table 2:**
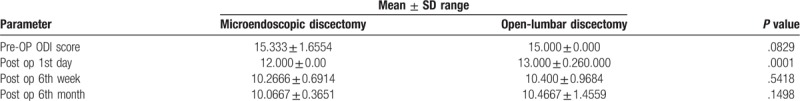
ODI scores for the patients in each group.

**Table 3:**
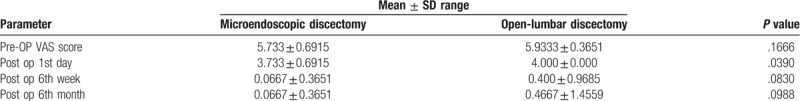
VAS scores for the patients in each group.

## 6 Discussion

LDH almost always occurred by the degeneration of the nucleus pulposus and annulus fibrous from the intervertebral disc, especially when they compress on the nerve root, which is the major cause for lower back pain. The MED is an approach of choice for the compressed nerve root and the herniated disc. It is less traumatizing to the paravertebral muscles, results in less fibrosis inside the canal and less morbidity, shorter hospital stay, better VAS, and ODI score and greater overall patient satisfaction. Currently, in our study, the surgical time for MED was shorter in most of the cases. The mean surgical time was 84 minutes, which is shorter than 106 minutes in another study of 25 patients treated by MED. Most studies on microdiscectomy and percutaneous discectomies report a surgical time of 40 to 120 minutes.^[37–39]^ In our present study, hospital stay was significantly shorter in patients having MED than open discectomy (10.63 vs 22.33 days, *P* < .0473), consistent with a study comparing MED to Love method (8 vs 24 days).^[40]^ The shorter period of postoperative disability may be attributed to the absence of the epidural fibrosis and tethering of nerve roots that commonly ensue after laminotomy.^[41,42]^ The epidural venous systems are not disturbed during MED. This helps to prevent venous stasis and chronic nerve-root oedema. The minimum surgical trauma inflicted on myoligamentous structures may facilitate rapid recovery. Also, it does not entail traumatic nerve root dissection, extra bone removal or large skin incisions. The risk of complications from scarring, blood loss, infection, and anaesthesia is considerably reduced or eliminated. The patient demographics and outcomes parameter Mean ± D (range), *P* value of microendoscopic between open discectomy age (years) 57.50 ± 7.63 (22–90) and 58.26 ± 1.44 (37–74), no. of male:female 15:15 and 14:16, surgical time (minutes) 84.00 ± 1.59 (45–195) and 199.83 ± 1.21 (80–200) *P* < .006. Anesthesia used in MED is local whereas, for OLD general anesthesia is used, there is more chance of anesthetic complication, but during the time period of our study complication was not encountered. Blood loss (ml) 14.00 ± 2.20 (10–50) 626.66 ± 8.98 and (100–1500) *P* < .0001 10.63 ± 4.09 (7–22) 22.33 ± 7.93 (16–48) < 0.047 ∗ *P* = .05 at baseline, which is approximately less and similar to others study.^[53]^

In this study, the VAS and ODI scores were used for clinical effectiveness assessment. There are significant differences between the VAS and ODI scores of the 2 groups on day first postoperatively which is clinically and statistically significant (*P* < .05). In comparison the score at 6th weeks and 6th months postoperatively in both groups, the result was clinical improvement significant in each group but statistically not significant (*P* > .05). Time mean Oswestry score microendoscopic discectomy and open discectomy Preop∗ 15.33:15.00 Postop 1st postoperative day 12.00:13.00, 6weeks 10.26:10.40, 6th months 10.60:10.46. Whereas, time mean VAS score microendoscopic discectomy and open discectomy Preop∗ 5.73:5.93, Postop 1st postoperative day, 3.73:4.00, 6 weeks 0.06:0.40, 6th months 0.066:0.066. Which shows that there was improvement in Oswestry and ODI score in MED and OLD in both groups as well.

Kulkarni et al^[52]^ reported a prospective study of 188 consecutive patients who underwent surgery for herniated disc using the tubular retractors between April 2007 and 2012. All patients had a preoperative MRI (Magnetic Resonance Imaging) and were operated by a single surgeon with the METRx system (Medtronic, Sofamor-Danek, Memphis, TN) using 18 and 16 mm ports. All patients were mobilized as soon as pain subsided and discharged within 24 to 48 hours post surgery. The results were evaluated by using VAS (Visual Analog Scale 0–5) for back and leg pain and ODI (Oswestry Disability Index). Patients were followed up at intervals of 1 week, 6 weeks, 3 months, 6 months, 12 months, and 2 years. In his study result was found that the mean age of patients was 46 years (range 16–78 years) and the sex ratio was 1.5 males to 1 female. The mean follow-up was 22 months (range 8–69 months). The mean VAS scale for leg pain improved from 4.14 to 0.76 (*P* < .05) and the mean VAS scale for back pain improved from 4.1 to 0.9 (*P* < .05). The mean ODI changed from 59.5 to 22.6 (*P* < .05). The mean operative time per level was about 50 minutes (range 20–90 minutes). Dural punctures occurred in 11 (5%) cases. Average blood loss was 30 ml (range 10–500 ml). A wrong level was identified and later corrected in a case of revision discectomy. Four patients with residual disc-herniation had revision MED and 3 patients with recurrent disc herniation later underwent fusion. One patient had a wound infection which needed a debridement. He concluded that MED for herniated discs effectively achieves the goals of surgery with minimal access. The advantages of the procedure are cosmesis, early postoperative recovery, and minimal postoperative morbidity. He et al^[51]^ reported 5 randomized controlled trials involving 501 patients were included in this meta-analysis. The pooled analysis showed that there was no significant difference in the VAS, ODI, or complication between the 2 groups. However, compared with the open discectomy, the microendoscopic discectomy was associated with less blood loss [WMD = −151.01 (−288.22, −13.80), *P* = .03], shorter length of hospital stay [WMD = −69.33 (−110.39, −28.28), *P* = .0009], and longer operation time [WMD = 18.80 (7.83, 29.76), *P* = .0008]. He concluded that MED, which requires a demanding learning curve, may be a safe and effective alternative to conventional open discectomy for patients with lumbar disc herniation.

The recent evolution of percutaneous endoscopic procedure as well as its current stance as a feasible treatment alternative for various lumbar spine pathologies. Reflecting the higher demands from the patients’ for a smaller incision, minimized manipulation to normal structures, as well as consequent faster recovery, these percutaneous procedures have been well acclaimed from various minimally invasive spinal surgery (MISS) related societies around the globe recently. Novel advances in the field of endoscopic optics and miniaturized but reinforced surgical tools would be thoroughly scrutinized and compared to assess their future capabilities to replace the conventional “open” surgeries under microscopic view. In the past 2 decades, minimally invasive spine (MIS) surgery has been increasingly applied and drawn much attention in the treatment of spinal disorders.^[45]^ To date, there has been a higher demand in patient's request to conduct this surgery, and the traditional open spine surgery has gradually been replaced with MIS surgery. According to the reports, the number of MIS instrumented surgeries conducted in 2010 accounted for 1/6 of the total number of all spine surgeries in the United States and 1/3 in 2016, which is anticipated to be more than 1/2 in 2020.^[43,44]^ With the aids of modern diagnostic and navigation technology, innovative spinal devices, and optical with improved MIS instruments, MIS surgery does how its merits including a smaller skin incision, less trauma to paravertebral soft tissues, reduced blood loss during operation, and a faster functional recovery in these patients, the same or even better outcome compared to traditional open spine surgery is still very limitedly elucidated. However, we are glad to see that these changes might lead to better patient surgical outcomes and reduce the economic burden^[46]^ for the medical cost related to postoperative hospital stay or complications. Over the past 10 years, the important role of percutaneous full endoscopic interlaminar/transforaminal surgery has been reassessed in patients with degenerative lumbar disc diseases or stenosis.^[47–50]^ This technique has been proven to work satisfactorily as other procedures even in patients with complex spinal degeneration or mild to a moderate deformity that is usually considered a reason for fusion surgery in most of our past surgeries. Furthermore, the full endoscopic interlaminar/transforaminal. Surgery has become a daily surgical practice in many spine centers around the world.

The major limitation of this study was that the number of cases was not large. We used a strict criterion for patient selection. However, another limitation was the difficulty in suturing dural tears properly due to limited room for suturing tools; second, a demanding learning curve to gradually trade the hand-eye coupling of the open surgical field with the two-dimensional view and hand-eye spatial separation of the MED procedure. The surgeon should be engaged with the senior surgeon in MED for observation and assistance plus attending workshops to practice on cadavers. And, we also did not comment on the subject of cost-effectiveness, which is a pity that it is a complex system. Besides, clinical heterogeneity may be caused by various surgical instruments and operative proficiency in different treatment centers. Finally, all of the documents were in English, there may be a language bias.

## 7 Conclusion

The standard surgical treatment of lumbar disc herniation has been open discectomy but there has been a trend towards minimally invasive procedures. MED for lumbar spine disc herniation is a well-known but developing field, which is increasingly spreading in the last few years. The success rate of MED is about approximately 90%. Both methods are equally effective in relieving radicular pain. MED entailed shorter hospital stay, less morbidity, and earlier return to work less anesthetic exposure less blood loss, less intra-op time comparing to OLD. MED is a safe and effective alternative to conventional open discectomy for the patients with lumbar disc herniation. But MED procedures need more skill and super specialized training to performed perfectly.

## Author contributions

**Conceptualization:** Ram Ishwar Yadav, Cao Yanming.

**Data curation:** Ling Long.

**Funding acquisition:** Cao Yanming.

**Methodology:** Cao Yanming.

**Supervision:** Cao Yanming.

**Writing – original draft:** Ram Ishwar Yadav.

**Writing – review & editing:** Ram Ishwar Yadav.
